# Goitre in Liberia

**Published:** 1876-04

**Authors:** 


Goitre in Siberia.—Goitre prevails only to a slight extent in Russia in Europe, its vast plains offering few facilities for its development. It is, however, met with in some isolated villages in some of the Governments. In Siberia, on the contrary, it prevails endemically. According to a statistical account published by Dr. Hachine, in a population of 365,810, in the Government of Irkoutsky, which is watered by the Lena and its affluents, there existed, in 1870, 3,400 subjects of goitre and 161 cretins. In some villages twelve and even twenty-five per cent. of the inhabitants were goitrous, and a Cossack regiment 5,040 strong furnished 436 instances of goitre. The affection, it is said, was not known in Siberia previous to the Russian conquest, and is chiefly due to the accumulation of filth and manure in the deep, unventilated valleys where it prevails. Its presence is much favored by the habit of the Russian peasants of keeping their abodes perfectly closed up, and which they have carried with them to Siberia. The indigenous inhabitants, who frequently change their places of abode, which are also better ventilated, are only exceptionally goitrous, and then only when they adopt the Russian mode of life.—*Jour. de la Societe de Statisque.*

